# Recent Developments and Applicability of In Vitro Gut Microbiota Models in Biomedical Research and Digestive Diseases—A Systematic Review

**DOI:** 10.3390/medicina62030554

**Published:** 2026-03-16

**Authors:** Ioana-Miruna Balmus, Gabriel Dascalescu, Viorica Rarinca, Alin Ciobica, Elena Toader, Georgiana-Emmanuela Gilca-Blanariu, Simona Stefania Juncu, Carol Stanciu, Anca Trifan

**Affiliations:** 1 CENEMED Platform for Interdisciplinary Research, Grigore T. Popa University of Medicine and Pharmacy Iasi, 700115 Iasi, Romania; balmus.ioanamiruna@yahoo.com (I.-M.B.); alin.ciobica@uaic.ro (A.C.); 2 Department of Exact Sciences and Natural Sciences, Institute of Interdisciplinary Research, “Alexandru Ioan Cuza” University of Iasi, 26th Alexandru Lapusneanu Street, 700057 Iasi, Romania; 3 Doctoral School of Biology, Faculty of Biology, “Alexandru Ioan Cuza” University of Iasi, Carol I Avenue, 20A, 700505 Iasi, Romania; gabidascalescu2001@gmail.com; 4 “Ioan Haulica” Institute, Apollonia University, Pacurari Street 11, 700511 Iasi, Romania; rarinca_viorica@yahoo.com; 5 Department of Biology, Faculty of Biology, “Alexandru Ioan Cuza” University of Iasi, Carol I Avenue, 700506 Iasi, Romania; 6 Academy of Romanian Scientists, 050094 Bucharest, Romania; 7 Department of Gastroenterology, Faculty of Medicine, Grigore T. Popa University of Medicine and Pharmacy Iasi, 700115 Iasi, Romania; toader.elena@yahoo.com (E.T.); simona.juncu@yahoo.com (S.S.J.); stanciucarol@yahoo.com (C.S.); ancatrifan@yahoo.com (A.T.); 8 Institute of Gastroenterology and Hepatology, “Sf. Spiridon” County Clinical Emergency Hospital, 700111 Iasi, Romania

**Keywords:** gut microbiota, in vitro models, advantages, limitations, applications, dynamic fer-mentation, batch fermentation, cell co-cultures, organoids, gut-on-a-chip

## Abstract

*Background and Objectives*: Current research approaches focusing on the human gut microbiota require complex in vitro systems that could provide sufficient viability and similarity with the conditions provided by the human intestine. As critical physiological functions, such as metabolic and inflammatory modulation, are associated with gut microbiota activity, complex host–microbiota interactions represent a pivotal new direction for therapeutic and nutritional interventions. However, there are several limitations to the current development of advanced in vitro models. *Materials and Methods*: A systematic review was performed according to the PRISMA guidelines for data collection and interpretation. *Results*: This manuscript summarizes the most advanced in vitro approaches for studying the gut microbiota, including batch fermentation models, dynamic fermentation models, and state-of-the-art technologies, such as organoids and gut-on-a-chip platforms. Each model offers beneficial study backgrounds, advantages, limitations, and the capacity to replicate the physiological complexity of the intestinal environment. However, due to the increased heterogeneity of the reported models, there is an urgent need for standardization. In this way, coherent regulatory frameworks are needed to guide the development and application of in vitro models. *Conclusions*: By consolidating knowledge and critically addressing current challenges, this study contributes to gut microbiota research by providing a direction for ethical, precise, and high-impact scientific studies.

## 1. Introduction

The gut microbiota is a collection of microorganisms colonizing the gastrointestinal tract and consisting of bacteria, viruses, archaea, fungi, and other eukaryotic microorganisms [1,2]. Most recently, gut microbiota has been shown to play essential roles in maintaining host health by protecting against pathogens, regulating homeostasis, and contributing to digestion and immunity [3,4].

While a close correlation between gut microbiota imbalances and numerous diseases has been emphasized, further research needs to fill the gap in defining the terms of this correlation (causation or consequence). In this context, various animal models have been designed to contribute to the understanding of the microbiota-health relationship [5,6]. Despite groundbreaking results, research using animal models could be heavily restricted by ethical constraints, high costs, and time constraints. Thus, a more sustainable alternative could be represented by in vitro models, which gained increasing popularity due to their marked advantages [7] and became fundamental tools in biomedical research, providing unique opportunities to analyze complex biological processes in controlled environments. Their ability to mimic the structural and functional characteristics of organs enables a deep understanding of the interactions between microorganisms, cells, and human tissues. Over the past decades, in vitro technologies have significantly evolved, consisting of cultures of primary cells, organoids, organ-on-a-chip devices, and advanced 3D bioengineering platforms. The variety of in vitro alternatives to animal models allows extensive coverage for multiple research directions by adjusting biological complexity levels at affordable costs and efficient implementation [7,8]. Similar to animal models, the standardization of in vitro models is essential in guaranteeing design reproducibility and applicability in molecular pathology or pharmacological contexts [9].

In this context, we aimed to investigate the potential of the most recent gut microbiota in vitro models in improving the current research approaches in the area of digestive diseases, in the current context of limitations due to the ethical, technical, and financial drawbacks of animal models.

## 2. Materials and Methods

We addressed our aim by conducting a systematic review based on a literature search using the most common research databases, such as PubMed, Scopus, Google Scholar, ScienceDirect, and Cochrane Database of Systematic Reviews. This systematic review was registered on the PROSPERO platform (CRD420251015746/20 March 2025). PRISMA guidelines were used to perform the data collection and interpretation (File S1).

The selection criteria included: (1) full-text research studies written in English or having an English translation available by the publisher; (2) published between 1990 and September 2025; (3) presenting data regarding in vitro gut microbiota models reported as efficient to study digestive diseases. Exclusion criteria included: (1) any paper that was not written in English or with available English translation on the publisher platform; (2) not available in full text; (3) any study falling within the following types: reviews, comments, letters to editors, preprints, and conference posters; (4) not presenting relevant information about the methodological establishment for the in vitro gut model or not being optimized for digestive diseases research.

Two distinct investigators conducted parallel searches. To ensure high specificity and reduce the retrieval of out-of-scope generalized microbiome literature, complex search strings were constructed using MeSH terms and Boolean operators. A representative search strategy applied in PubMed/MEDLINE was: ((“Gut Microbiota” OR “Gastrointestinal Microbiome”) AND (“In Vitro Techniques” OR “Organoids” OR “Lab-On-A-Chip Devices” OR “Batch Fermentation” OR “Dynamic Fermentation” OR “Co-Culture”) AND (“Digestive System Diseases” OR “Inflammatory Bowel Diseases” OR “Colorectal Neoplasms”)). A similar translated search string was adapted for Scopus, ScienceDirect, and Cochrane. The screening workflow consisted of removing duplicates, pre-screening titles and abstracts for direct relevance to the pre-defined triad (microbiota + in vitro model + digestive disease), followed by a full-text assessment against the inclusion/exclusion criteria. The studies were first pre-screened for title and abstract content. Following the exclusion of duplicates, the selection process was performed by considering the relevance, the novelty, and the quality of the findings. Unfortunately, we were unable to perform a meta-analysis of the data due to the high level of heterogeneity across the studies, at all levels, including patients’ demographics, study design, methodological approaches, and reported outcomes. In this way, the results of the current study are presented narratively.

## 3. Results

**Figure 1:**
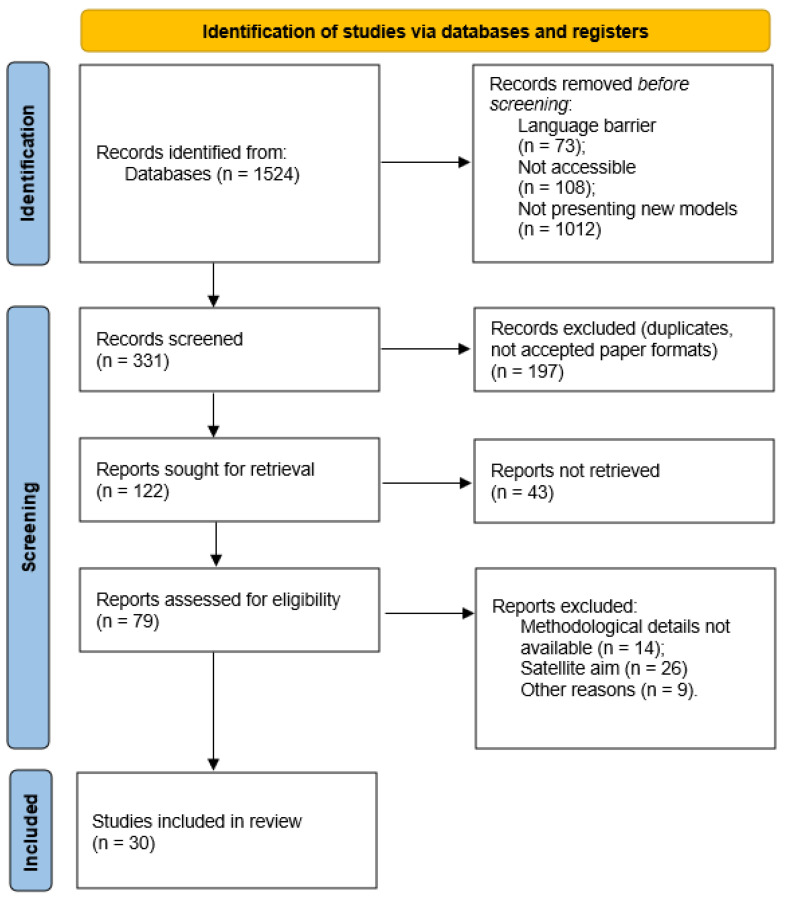
PRISMA flowchart.

The initial search using the search string on all the databases retrieved 1524 results (Figure 1). Language barriers and non-accessibility led to the exclusion of 181 results. The majority of studies were excluded due to reviews of literature or not presenting innovative in vitro models (i.e., studies that used previously validated models from other reports) (n = 1012). Thus, the retrieved number of studies (n = 331) reflects the highly specific and stringent nature of our research strategy. Rather than capturing the vast, generalized literature on the microbiome, our inclusion criteria intentionally required the intersection of three strict parameters: the use of distinct in vitro models, the availability of comprehensive methodological reporting, and a dedicated optimization for digestive disease research. This specific focus heavily filtered out generic microbiome studies to isolate high-fidelity models applicable to gastroenterology. Another 110 duplicates were excluded, while 87 studies were excluded due to comments, letters to editors, preprints, or conference posters. Another 12 studies were excluded due to an English translation not being available, while 43 studies were not available in full text. Another 14 studies were excluded due to not presenting full methodological details; 26 studies focused only secondarily on setting in vitro models for gut microbiota research, while 9 were excluded for other reasons. The remaining 30 were considered for the current systematic review. Table 1 summarizes the main in vitro model types used in the study of the gut microbiota, highlighting their role in elucidating microbial interactions, drug metabolism, and toxic transformations.

**Figure 2:**
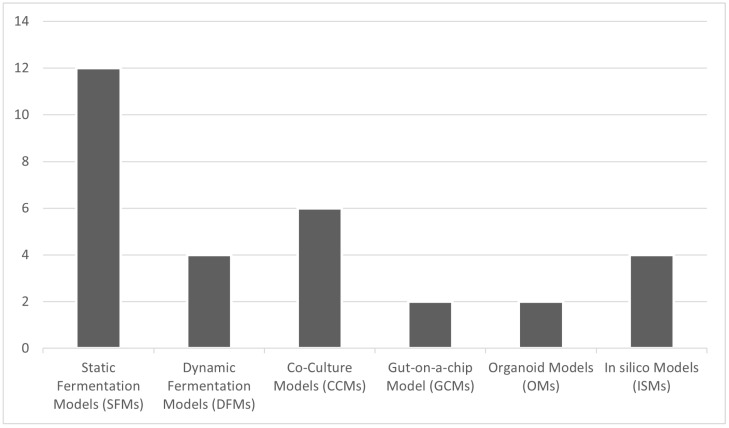
Distribution of the included high-fidelity studies (n = 30) categorized by the employed in vitro and in silico gut microbiota model type.

We analyzed the distribution of the 30 studies we selected based on the type of in vitro model employed to provide a clearer overview of the literature landscape (Figure 2). We found that 40% of the high-fidelity studies we selected presented SFMs, which are generally simple short-term microbial models [10,11,12,13,14,15,16,17,18,19,20,21]. Twenty percent of the studies described recognized CCMs [26,27,28,29,30,31], while less than 15% reported DFMs [22,23,24,25] and ISMs [36,37,38,39]. The most complex models were the least common, including GCMs [32,33] and OMs [34,35], which together accounted for less than 10% of the studies.

**Figure 3:**
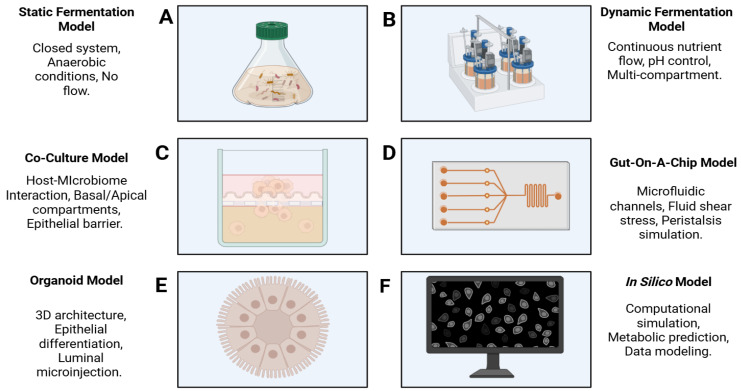
Consolidated schematic representation of the main in vitro and in silico models used for gut microbiota–host interaction research. (**A**) SFMs represent simple, closed anaerobic environments suitable for short-term microbial metabolism screening. (**B**) DFMs utilize multi-compartment bioreactors with continuous nutrient flow and pH control to simulate long-term gastrointestinal transit. (**C**) CCMs employ Transwell inserts to separate apical and basal compartments, allowing the study of interactions between the microbial community and the epithelial barrier. (**D**) GCMs integrate microfluidic channels to simulate fluid shear stress and continuous peristalsis. (**E**) OMs provide a 3D architecture with complete epithelial differentiation, often utilizing microinjection for luminal microbial colonization. (**F**) ISMs employ advanced computational algorithms and databases to predict community metabolic shifts and host–microbiome dynamics. (Image designed with Biorender.com).

This highlights the predominant use of static and co-culture methodologies in recent digestive disease research. A consolidated schematic comparison of these experimental setups, illustrating their structural complexity and key physiological features, is provided in Figure 3.

### 3.1. Static Fermentation Models

Static fermentation models (SFMs), also known as batch fermentation models, are a simple, accessible, and versatile method for replicating human gut microbiota [40]. While operating in closed anaerobic environments, SFMs allow short-term simulations, such as when assessing the effects of certain dietary components on the intestinal microbiota. For instance, Pham et al. [10] developed short-term microbial communities by setting up a static model using anaerobic conditions, nutritive media, and human feces-derived inoculum to evaluate the effects of various vitamins on bacterial growth and diversity. Similar setups were used to evaluate the influence of different microelements, prebiotics, and probiotic formulations in modulating microbiota diversity and metabolism [12,14,18]. Dash-Walsh et al. [11] developed SFM to study the bacterial metabolism of trimethylamine (TMA), an important precursor of TMA N-oxide that is associated with a significant risk for chronic pathogeneses, such as cardiovascular and metabolic diseases. Although the model was designed to focus on metabolic outputs of the microbial cultures derived from fecal inoculum from healthy donors, the study suggested that it could be a valuable research solution for evaluating the possible modulators of TMA metabolism within the human intestine. Similarly, Goya-Jorge et al. [13] used controlled nutritive media enclosed in 600 mL glass flasks connected to an anaerobic system to ferment fecal inoculum from healthy donors to simulate a short-term microbial culture system (up to 72 h) to study the influence of bacterial metabolites on the host transcription factor activation that contributes to immunological response. The model has great potential in analyzing the metabolic processing of xenobiotics, yet the static nature of the design could limit the physiological relevance.

SFMs could not only be designed with fecal inoculum but also with standardized strains that could enable the research of probiotic contribution to gut microbiota. In this way, Das et al. [17] used standard strains (*Bifidobacterium adolescentis, Bacteroides thetaiotaomicron, Faecalibacterium prausnitzii,* and *Roseburia inulinivorans*) to simulate a short-term (up to 96 h) SFM for designing novel in vitro pair combinations with relevance to short-chain fatty acids metabolism (*B. adolescentis* and *B. thetaiotaomicron, F. prausnitzii*, and *R. inulinivorans*, respectively) of beneficial human gut bacterial species based on co-occurrence network analysis of large metagenomic datasets. Bacterial abundance in relation to competition for the provided resources or produced secondary metabolites, the characterization of extracellular metabolites, and the influence of pH on these were the main outcomes of this study. However, the synthetic character of the formulated bacterial community and the reductionist approach that prevented the observation of effects of fluctuating conditions and resource availability could limit the applicability of the model in studying the implications of microbial factors in disease susceptibility.

Toxicity studies also used SFMs to evaluate the toxic potential of various compounds on the gut bacterial communities. For example, Kollarczik et al. [19] studied the effects of mycotoxins obtained from *Fusarium* fungi. Static bacterial cultures derived from pig fecal inoculum were used to simulate intestinal fermentation in short-term controlled nutritive media (24 h). The advantages of such SFM include an inexpensive and simple way to evaluate mycotoxin biotransformation during digestion in mammals. Yet, the applicability to more complex studies is limited and could necessitate improvement in addressing other research perspectives, such as bacterial transformation product cytotoxicity. Pharmacological approaches also used several SFMs to evaluate the effects of the biotransformation of different drugs by intestinal microbiota. For instance, Guo et al. [21] used non-alcoholic steatohepatitis mouse model-derived short-term (72 h) microbial communities to describe the metabolism of omeprazole, phenacetin, midazolam, tolbutamide, metoprolol, and chlorzoxazone.

SFMs were also used to simulate and compare pathological conditions with healthy intestinal environments. Gonza et al. [16] designed a short-term (72 h) static SHIME model to study the differences between normal and IBD-related digestive responses to food additives. However, despite the model offering a milestone in this area of research for being able to simulate gut bacteria cultures obtained from both healthy and IBD-diagnosed donors, with the possibility of a standardized protocol to assess the impact of food additives on microbial diversity and immune response to metabolites in bacterial communities, the important limitation of individual variability of gut microbiota remained. Another example relevant to healthy versus pathological conditions is the study of Maccaferri et al. [20] that described a multisegmented SFM corresponding to colonic segments. The purpose of this model was to evaluate the potential beneficial effects of the non-absorbable antibiotic, rifaximin, on the diversity and metabolism of the microbial communities within the intestines of colonic active Crohn disease patients, contributing to the various reports’ conclusions regarding the efficiency of rifaximin to induce remission in IBD [20].

More complex approaches of SFMs include the simulation of multiphase digestion produced by microbial communities residing in different compartments of the human digestive system. Brodkorb et al. [15] elaborated a standardized protocol for simulating multicompartment food digestion in static conditions. Each phase consisted of nutritive media enriched with simulated digestive fluids (simulated saliva, gastric fluid, or intestinal fluid), digestive enzymes (amylase, pepsin, gastric lipase, pancreatin), together with inorganic and bile salts. However, the authors identified several key limitations when thinking of translating to pathophysiology. For instance, the normal flux of the gastrointestinal tract could not be simulated by this model, as the static character prevented obtaining the gradual addition of digestive fluids, gastric emptying, nutrient-modulated digestive enzymes secretion, and sequential intestinal digestion. In this way, this SFM could be more suitable for studying digestive endpoints rather than digestive kinetics.

However, the applicability of these models is restricted to studying the behavior of microbial cultures in relation to different external interventions, such as changes in nutritional substrate composition or in species diversity, not accounting for the specific traits of the intestinal environment or dynamics. Also, static models are characterized by growth inhibition related to substrate consumption or toxic metabolites accumulation [41]. Despite that, SFMs’ designs widely vary, considering the range of the simulation complexity, from sealed flasks (inoculated with unique or mixed microbial species) to controlled reactors (fecal suspensions) [42], they are limited in reproducing the dynamic complexity of the living intestinal environment [10,11,18,19].

Crucially, besides failing to recapitulate dynamic aspects of intestinal physiology, such as continuous nutrient absorption, the physical barrier of the mucus layer, and mechanical motility, SFMs fail to comply with studies of the host–microbiome interaction. However, they remain highly valuable for initial screenings. SFMs are also suitable for evaluating the digestibility and bioaccessibility of various compounds, including drugs [20,21], mycotoxins [19], and the release of micronutrients, such as plant secondary metabolites (e.g., carotenoids or polyphenols) [43]. A possible solution to this important limitation could be the combination of SFMs with other research solutions. Huangfu et al. [44] have recently studied the effects of gut microbiota on dietary fibers and possible metabolic significance for the host in both in vitro and in vivo models. The combination of in vitro and in vivo was argued as the initial evaluation of SFMs resulted in the partial observation of short-chain fatty acids and gas production, thus mimicking intestinal fermentation. However, while the gastrointestinal tract dynamics are complex, a complete picture could be reached by using animal models as an observational environment [44].

### 3.2. Dynamic Fermentation Models

More complex in vitro models were developed for long-term studies of microbial metabolism, adaptation, and ecology. In this context, the dynamic fermentation models (DFMs) could provide a better understanding of these aspects by allowing extended timespans of observation by substrate replenishment and toxic products filtration [7,45]. Thus, designing more complex studies that included the understanding of microbiota changes during digestion was enabled by the standardization of the SHIME system [23].

In this way, one of the most efficient DFMs, the SHIME system, was designed to simulate digestion using multichambered bioreactors and peristaltic pumps [23]. Moreover, it could provide a constant pH to model pancreatic and bile secretory functions and real-time gastrointestinal transit. The model was initially developed using suckling piglet fecal inoculum [23], but was later standardized for human feces [25]. Thus, DFMs offer a net advantage in being able to simulate condition variability and microbiota–host interaction complexity from the intestinal environment [7]. As the in vitro models of DFM are constantly fed with essential nutrients by peristaltic pumps, more complex ecosystems are also possible to obtain [42]. Furthermore, the development of MiPro culture media and the SHIME system was an important milestone for in vitro model research, as both were validated for animal and human-originating inoculum [23,25].

By contrast to the static SHIME model, the dynamic one provided technical background for longer observation periods (up to 4 weeks) and the possibility to address serial transformations in multi-chambered bioreactors that could successfully simulate mammalian gut structure and functions, including peristalsis, pancreatic juice, bile, fermentation liquids, and constant pH. However, the authors mentioned that the standardization focused on mammalian gut microbiota other than human, which is an important limitation of this promising DFM [23].

Another important milestone in DFM’s development was the description of the standardization of human real intestinal content and feces inoculation in the most complex and costly SHIME peristalsis system, resulting in a validated DFM for distal small intestine fermentation [25]. Despite the limited capacity to simulate host–microbiome interactions, MiPro-based DFM was reported to be fit for functional and compositional studies of intestinal microbiota.

Another relevant example could be the DFM reported by Gibson et al. [46] in which a mixed culture of human fecal bacteria was grown for 120 days in a three-stage continuous culture system. The comprehensive technical system that assisted in accurately reproducing the essential nutritional needs and pH consistency included advanced growth systems with constant pH control and monitoring of key parameters, such as hydrogen production, methane, sulphate reduction, and volatile fatty acid production. Successive controlled growth environments were used to simulate the nutrient and metabolite flow and gradients characteristic of the cecum, transverse, and descending segments of the colon [46,47]. By maintaining this continuous culture for 120 days, the model generated key insights into the spatial distribution of bacterial metabolism, specifically demonstrating regional variations in volatile fatty acid production that closely mirror in vivo conditions. Smaller-scale DFMs, such as the CoMiniGut prototype, are also useful to evaluate gut microbiota metabolism in more complex experimental conditions, as compared to SFMs [24]. In this way, DFMs were consistently described as efficient in vitro models to study intestinal microbiota participation in host digestion. Moreover, both SFMs and DFMs, such as MiPro and SHIME [22,23,25], demonstrate the ability to replicate dynamic, functional, and compositional gut microbiota with high fidelity, often correlating with in vivo results.

### 3.3. Co-Culture Models

Co-culture models (CCM) were initially developed to allow the study of the interactions between the host cells and gut microbiota [7]. The effects of dietary components on intestinal epithelial cells and immune cells were extensively evaluated and described by using comprehensive CCMs [28,29,30,31]. For instance, Xiang et al. [28] established a three-stage colon simulation containing mucosal beads to demonstrate how xylitol supplementation directly enhances propionate synthesis through microbial cross-feeding. Similarly, Wang et al. [29] utilized CCMs to reveal the immunoactivation pathways triggered by fucoidan fermentation products, generating key insights into diet-microbiome-host interactions. Moreover, they used Caco2 cell lines as a background for their CCM and co-cultured murine macrophage RAW264.7 to evaluate the potential of murine feces-derived fermentation products of fucoidan to modulate intestinal inflammatory response.

However, due to the short-time-span designs and low complexity of the controlled environments, the applicability of CCMs remains limited. Furthermore, while incorporated host cells, CCMs often lack functional vascularization, endogenous bile acid signaling, and the complex mechanical cues required for complete epithelial differentiation, which may alter the interpretation of immunological response. Yet the most important advantage of CCMs resides in providing sufficient growth conditions for anaerobic intestinal species and an aerobic environment for the intestinal epithelial cells [48]. The use of these systems is proven by the ability to accurately mimic the microbiota–host interactions [48]. For instance, Caco-2 cell lines are hybrid models integrating in vitro and in vivo technologies to provide valuable insights into microbiota-drug interactions, highlighting their implications for therapeutic efficacy and pharmacokinetics [27,30]. The use of CCMs based on Caco2 cell lines was an important milestone in drug pharmacokinetics research, as Degraeve et al. [30] described the design of a potential pharmacokinetic CCM that could enable drug dosage prediction. Most frequently, CCMs use Caco2 cell lines to simulate the functionalities of mature enterocytes, including tight junctions and microvilli [27], and are often combined with other cell lines to obtain high-fidelity simulations of the intestinal lining. A recent study designed a CCM using Caco2 cell lines alongside HT-29 and HMC-1.2 cell lines to obtain linings of mature enterocytes, mucus-secreting goblet cells, and mast cells that simulate the intestinal mucosal barrier. In this context, probiotic formulations (based on standardized strains of *Lactobacilli* and *Bifidobacterium*) were used to evaluate modulatory traits in inflammatory response, making CCMs great candidates for testing therapeutic approaches in gut inflammatory diseases. In other model designs, cell lines could not be the main interest. For example, Gratz et al. [31] designed a CCM based on human Caco2 and TC7 cell lines to simulate intestinal lining with functional cellular transport, permeability, apical brush border, and high hydrolase activity, which are all crucial in studying intestinal content metabolism during digestion, such as mycotoxins.

Furthermore, these models demonstrated the ability of the microbiota to transform xenobiotics and mycotoxins into less harmful metabolites, highlighting their potential in assisting toxicity and bioavailability studies [29,31]. Despite their multiple advantages, the studies reported many environmental setups, and thus rigorous standardization should provide reproducibility and broader applicability [30,31]. A particular case of host–microbiota interaction in the context of gut microbiota contribution to gut inflammatory response in the pathophysiology of spondylitis was described by Beterams et al. [26] when three different types of human cell lines were used to establish the CCM. In this case, T84, LS-174 T, and THP-1 cells all contributed to designing a simulated intestinal lining consisting of intestinal epithelial cells, goblet cells, and macrophages.

### 3.4. Gut-on-a-Chip Models

The inability of less complex models to mimic an entire organ was overcome by a complementation with more performant technologies, such as the gut-on-a-chip paradigm [49,50,51]. Gut-on-a-chip models (GCMs) are advanced microfluidic systems that manage to recreate the complex environment of the colon in a highly realistic way. These systems could integrate multiple environmental components of the human intestine, including fluid flows, nutrient gradients, and microbiota–host interactions [50,52] to provide an ideal platform allowing the observation of gut microbiota in relation to the fed dietary components [53]. By being miniature colons that mimic both the structure and functions of the epithelial layer of the intestinal lining, GCMs are a complementary solution to CCMs in studying the intestinal environment dynamics [33]. In this way, microdevices that include microchannels of cell cultures, vacuum chambers, and microfluidic cultures form a complex, mechanical miniaturized system that could simulate intestinal lining villi formation and intestinal peristalsis. A clear advantage of this complex model is that it could offer a miniature environment very similar to intestinal content and functions that could be used to study bacterial overgrowth and subsequent inflammatory response. Despite these net advantages, the model requires extensive resources and time for cells and microbial cultures.

Shear stress and peristaltic movements could be successfully mimicked by GCMs, leading to relevant data regarding the cellular responses to different pathophysiological conditions [54]. For example, Kim et al. [32] utilized a microfluidic gut-on-a-chip to demonstrate that mechanical deformation is crucial for intestinal homeostasis. The inter-relation between mucosal barrier integrity and bacterial capacity to adhere to the mucous layer was demonstrated using in vitro human colon monolayers and standard microbial strains (*B. thetaiotaomicron*). However, the fragile stability of the bacterial cultures in these environmental conditions remains an important limitation of this GCM that could correlate spontaneous bacterial overgrowth with host inflammatory responses, the latter being impossible to capture in SFMs or DFMs. In contrast to those, GCMs also provide combined fermentation conditions (both aerobiosis and anaerobiosis).

### 3.5. Organoids

Organoid models (OMs) are three-dimensional structures obtained by treating pluripotent stem cells to recreate complex intestinal structures [55]. The intestinal barrier physiology and other cellular processes involving the complex environmental conditions of the human intestine are the most common applications of OMs [56]. Fofanova et al. [35] successfully used standardized human intestinal organoids to obtain a close-to-reality in vitro condition to evaluate the effects of oxygen in modulating bacterial diversity. In this way, an oxygen gradient was set depending on the organoid architecture (apical—anaerobiosis, basal—aerobiosis) to study the host–microbiome interaction. However, the authors acknowledge the low reproducibility of the results due to fastidious culture conditions.

On the other hand, the most important advantage of OMs is the variability of architectural designs that could successfully be adapted to the numerous physiological and pathological conditions that are encountered in clinical practice and research [57]. Nigro et al. [34] thoroughly described the methodology to obtain close-to-reality simulations of intestinal crypts starting from mouse-originating small intestine samples, generating key insights into how specific bacterial products drive metabolic impairments during infection and alter the host’s inflammatory response. From a physiological perspective, while OMs provide excellent epithelial differentiation and structure, they inherently lack continuous peristaltic motility, endogenous vascularization, and a dynamic flow of luminal contents (including bile acid signaling). These limitations heavily restrict the stimulation of complete nutrient absorption and large-scale, long-term host–microbiome interactions.

Cellular matrices are usually the most efficient biochemical support for OM growth and development in studying infections, chronic diseases, and malignant processes. Thus, some OM applications could provide significant support for pharmacological and toxicological studies in conditions of replicated human physiology [58]. In this way, the host–microbiota interactions could be observed in OMs that facilitated the understanding of the microbiota’s influence on the host in aspects of health and disease [59].

### 3.6. In Silico Models

By contrast to the other models, in silico models (ISMs) are computational simulations allowing the prediction of host–microbiota interactions [60]. ISMs usually consist of complex databases that include information about microbiota, stimuli, and host characteristics that are combined by prediction algorithms [61]. Despite the disadvantages of not being tangible models and the difficulty of translation to biological systems, ISMs offer unlimited possibilities and setups for predictions. Thus, the potential to run complex simulations that involve huge amounts of data, such as metagenomic data, is a recognized trait of ISMs. In this context, Goris et al. [37] described an ISM that provided accurate bioinformatic analysis of genes and enzymes responsible for flavonoid transformations acting as possible microbiome-targeted therapies.

Crucially, because ISMs are entirely computational, they completely fail to physically recapitulate structural intestinal physiology, such as the mucus layer, physical immune components, mechanical motility, and spatial epithelial differentiations, making their outputs heavily reliant on in vitro or in vivo experimental validation. For instance, ISMs could offer simulations for single-compartment [39] or whole digestive tract digestion [62]. The simulation includes providing relevant input data to generate predictions or interpret causal relationships in biological systems. In this way, Henson et al. [36] used computational analysis to simulate computational models of microbial cross-feeding and evaluate the interdependence between the bacterial species of the human gut. Despite the advantage of fast and complex simulations of bacterial community stability and gut ecosystem resilience to microenvironmental changes, the translation of this ISM to biological systems, as well as of other ISMs, remains limited.

However, the validation of ISMs is rather laborious and requires in vivo or in vitro analyses. For instance, Marcheze et al. [38] used computational docking to model an ISM for studying the bioavailability and efficacy of antioxidants. Despite the promising results for nutraceutical development and therapeutic antioxidants research, validation with an antioxidant assay was mandatory, and the model remains limited in terms of translation to the physiological system. Despite these limitations, an important milestone in developing ISMs was reported when computational models provided valid resources in simulating pathological conditions associated with gut microbiota. Despite not being validated in clinical settings, the ISM described by Marcheze et al. [38] could elaborate the metabolic pathways of dysbiosis and could estimate microbiota modulation strategies for therapeutic interventions.

In this way, ISMs could generate information on the bacterial community dynamics, diversity, and metabolism during normal digestion [36]. For example, returning to the ISM described by Henson [39], their in silico metabolic model could successfully predict byproduct cross-feeding between specific bacterial strains, generating vital insights into community resilience and targeted metabolic shifts during dysbiotic pathological conditions. Another significant contribution of ISM technology in gastrointestinal tract research consists of developing fast and complex analyses on the possible bioavailability and metabolism of different compounds, such as antioxidants [37,38]. ISMs are, thus, powerful digital tools that contribute to identifying mechanisms and developing hypotheses for further experimental studies [63].

## 4. Discussion

During the last 35 years, in vitro models for gut microbiota have been used to raise the understanding of interaction mechanisms between particular compounds, such as food matrices, active pharmaceutical ingredients, or food constituents, and gut microbiota. As described, in vitro models of gut microbiota could be classified into static fermentation models, dynamic fermentation models, co-culture models, organoids, organ-on-chip, and in silico models [1,2,3,4]. Due to the growing interest in science and technology advances, in vitro models have increasingly begun to mimic in vivo intestinal topography and conditions, thus effectively providing alternatives to animal models [64]. The methodology of obtaining in vitro models offers several key advantages, such as enabling the observation of various cellular responses to external stimuli, proliferation, and viability [1] (Table 2). In this context, the relationship based on the mutual relationship between the host intestinal cells and gut microbiota, as well as the impact of different dietary components and the investigation of the intestinal microbiota’s implication in the metabolic processes, could be thoroughly studied. However, the studies we had selected for analysis pointed out that each model could be used to address different aspects of the interaction between diet, microbiota, and host, while having specific advantages and limitations.

The implementation of standardized in vitro models for studying host–microbiota interactions in intestinal diseases is dependent on several factors that are bound to the variability of human microbiota, the complex environment of the human gut, and the delicate balance between host tissues and the microbial community. Therefore, achieving reproducibility and biological relevance in these systems remains a persistent difficulty. Moreover, each intestinal disease is characterized by particular pathophysiological mechanisms that may contribute to complementary conditions and needs of the in vitro system.

### 4.1. Limitations of In Vitro Models for Inflammatory Bowel Disease

Several in vitro models have been developed to facilitate the research of the host–microbiota interaction in inflammatory bowel disease (IBD). The variants of IBD, Crohn’s disease, and ulcerative colitis are both characterized by chronic inflammation of the gastrointestinal tract and deficient gut microbiota functions [65]. While previous studies have described the complex interactions between genetic, environmental, immune, and microbial factors in IBD, it was shown that the host–microbiota interaction plays a pivotal role, as disruptions in the gut microbiome are linked to the onset and progression of intestinal inflammation [54].

As described before, the development of standardized in vitro models of host–microbiota interactions in the context of intestinal diseases faces considerable challenges, including the replication of the complex cellular architecture of the gut, simulating the dynamic and multifaceted gut environment, and, most importantly, capturing the intricate immune responses to microbiota [66].

The multilayered structure of the intestinal mucosa, which includes epithelial cells, immune cells, and a mucus layer, interacts with the resident microbiota [67]. Conventional in vitro systems, such as static culture systems, allow limited complexity, as compared to advanced models, such as organoids and gut-on-a-chip platforms with greater structural and functional fidelity. However, their high cost, advanced technical expertise requirements, and lack of standardization limit their applications [68].

Native gut microbiome reproduction for in vitro microenvironments could also face major limitations. The gut microbiota mainly comprises anaerobic species that require specific nutrients and pH levels provided by the host. In this way, the diversity and functionality of gut microbial communities are difficult to sustain under standard conditions. While this important limitation was partly surmounted by combining anaerobic culturing techniques and co-culture systems to improve microbial viability [69,70], the long-term interactions within the microbial community are still a technical challenge. To overcome this, emerging methodologies, particularly advanced microbial cultivation techniques (e.g., culturomics), can significantly enhance model utility. By expanding the repertoire of cultivable fastidious and strictly anaerobic taxa, these advances allow for the reconstruction of highly representative synthetic communities, improving reproducibility and allowing for the mechanistic validation of host–microbiome interactions. Based on the contribution of the immune system in the pathogenesis of IBD and in the gut response to microbial stimuli, incorporating immune components into in vitro models could be a significant step forward in reproducing the complexity of immune signaling and cellular interactions in in vitro models of IBD. While co-culture systems that include intestinal bacteria species, immune cells, and epithelia have shown promise, these setups often lack scalability and reproducibility [70]. Also, the lack of standardization across in vitro models, methodological variations, a wide range of cellular sources, microbial inoculum, and environmental parameters could lead to significant data inconsistencies and limit comparative analyses. In this context, microfluidic devices, advanced imaging techniques, and machine learning can enhance the accuracy and reproducibility of in vitro models for IBD. Additionally, integrating patient-derived cells and microbiota samples may help in personalizing models for better translational relevance.

The development of novel therapeutic approaches is also critical in IBD research. Thus, the in vitro model standardization should address the mechanisms underlying microbiota-modulated intestinal inflammation. While current models provide valuable insights, complexity, microbial viability, immune incorporation, and reproducibility require improvements. For instance, organoids using patient-derived tissue provide a promising resource in modeling microbiota–host interactions in native inflammatory conditions [34]. Recent studies have described the integration of patient tissue-derived organoids with microbial co-culture systems in studying personalized responses to dysbiosis [35]. Similarly, gut-on-a-chip technology simulates intestinal physiology with high precision, incorporating flow dynamics and oxygen gradients, which could also be significant contributions to IBD in vitro models.

### 4.2. Limitations of In Vitro Models for Colorectal Cancer

Significant microbiota–host interactions have been described in colorectal cancer (CRC) development and progression [71]. In this way, it was shown that the intestinal microbiota is intricately involved in colorectal carcinogenesis, influencing inflammation, immune modulation, and metabolic processes [72]. Moreover, dysbiosis could lead to carcinogenic metabolite production and exacerbated inflammatory response, both contributing to tumorigenesis [73]. In this context, the standardization of in vitro models for colorectal cancer should replicate the physiological and pathological microenvironments of the affected tissues, including pH, oxygen gradients, and nutrient availability [74]. Thus, developing co-culture systems including microbial species and viable tumor epithelial cells could be a significant contribution to current research efforts, yet remaining a challenging technical issue [75]. For instance, a two-way susceptibility between microbial overgrowth and host cell viability was described [76,77,78]. Other significant challenges that could contribute to preventing the development of efficient in vitro models for CRC could be the origin of microbiota samples and cell lines, and microculture protocols. Moreover, standardized guidelines for experimental parameters and conditions are lacunar, thus leading to inconsistent results. Also, similar to IBD, most in vitro models of CRC lack immune cells inclusion, thus limiting their applicability in microbiota-based host immunity modulation [79]. While more advanced models, such as OMs or GMs, require specialized equipment, expertise, and significant financial resources, promising results have been reported [80,81,82,83], and the need for global standardization protocols for microbiota preparation, culture conditions, and endpoint measurements is consistent across the studies.

### 4.3. Limitations of In Vitro Models for Disorders of the Gut–Brain Interactions

The most common disorders of gut–brain interactions that are currently under consistent research are irritable bowel syndrome (IBS), functional dyspepsia, and chronic constipation. These conditions are mainly characterized by altered gut motility, visceral hypersensitivity, and psychosocial factors that disrupt normal gut–brain signaling [84]. Moreover, recent studies showed that dysbiosis is an important component of the brain–gut interaction disorders [85,86,87]. However, the multifactorial character of these interaction modulations requires complex in vitro environments and specific conditions that remain significantly challenging. For instance, the brain–gut bidirectional interaction puts together neural, hormonal, and immune signaling, as well as secondary metabolite-based signaling excreted by the microbiota [88,89]. Thus, complex in vitro models should be developed to capture multiple pathological components in order to efficiently mimic the dynamic and reciprocal interactions between the gut and brain [89,90].

Promising results have been recently reported for several gut-on-a-chip or microbial co-cultured models, which managed to partially mimic microbial diversity, spatial distribution, and metabolic interactions of the human intestines [91]. On the other hand, functions such as intestinal peristalsis, mucus production, and a distinct oxygen gradient are much more challenging to obtain and remain future perspectives in this area. Another important limitation of in vitro models of the brain–gut interaction disorders is the lack of multiple cell types, such as epithelial cells, immune cells, and enteric neurons [7]. Various experimental designs and environmental conditions, microbial variability, and host cell lines could also lead to controversial results. In this context, the need for standardized protocols should be emphasized once more. Complex approaches, including enteric neuron modulation via microbial metabolites, could provide valuable future perspectives.

## 5. Conclusions

Extensive evidence has suggested a close correlation between gut microbiota imbalances and numerous diseases. Despite being a groundbreaking technical advance in research, animal models of gut microbiota–host interactions could be heavily restricted by ethical constraints, high costs, and time constraints. In this way, efficient and modern in vitro models became fundamental tools in biomedical research, providing unique opportunities to analyze complex biological processes in controlled environments. However, standardizing in vitro models to study host–microbiota interactions in gastrointestinal diseases remains challenging, while technological innovations and collaborative efforts are made to improve and innovate the current in vitro systems for both basic and pharmacological research. The integration of recent advances in bioengineering, computational modeling, and microbiological cultures could contribute to overcoming the current gaps and challenges in studying host–microbiota interactions.

## Figures and Tables

**Table 1:** Comprehensive overview of recent in vitro models of gut microbiota.

In Vitro Model	Experimental Design	Objectives and Milestones	Applications	Limitations	Ref.
SFMs	• Fecal inoculum from healthy donors; • Anaerobic conditions; • Supplementation with vitamins (A, B2, C, D3, E, B2+C, folic acid); • Sampling at 48 h.	• Developing fecal-derived short-term microbial communities to evaluate the effects of various vitamins on bacterial growth and diversity.	• Simple setup, cost-effective models microbial composition and metabolic activities.	• Limited simulation of gut microbiota and intestinal environment.	[10]
	• Fecal inoculum from healthy donors; • Anaerobic conditions; • Supplementation with choline, betaine, l-carnitine, γ-BB; • Sampling at 0, 24, 48 h.	• Developing a viable fecal-derived microbial community to study bacterial metabolism of trimethylamine (TMA). • Potential screening tool for pharmacological targets against intestinal TMA production.	• Controlled conditions for substrate analysis, focused on metabolic outputs.	• Lack of physiological complexity.	[11]
	• Fecal inoculum from healthy donors; • Anaerobic conditions, pH-controlled nutrient media; • Supplementation with an iron chelation agent; • Sampling at 0, 4, 8, and 24 h.	• A more complex BFM with high-fidelity nutritive media and controlled pH at physiological temperature. • A chelation agent was used to capture iron from the inoculum and study the effects of its absence on the bacterial community.	• Understanding the modulation of bacterial growth by iron.	• Does not account for host interactions.	[12]
	• Fecal inoculum from healthy donors; • Anaerobic conditions, pH-controlled nutrient media; • Sampling at 0, 24, 48, and 72 h.	• Efficient fecal-derived microbial community fit for bacterial diversity and bacterial metabolism studies. • Obtaining bacterial metabolites for further testing of human transcription factor activation.	• Understanding the impact of bacterial metabolites on the host immune defense and the metabolic processing of xenobiotics.	• Static nature may limit physiological relevance.	[13]
	• Fecal inoculum from healthy donors; • Anaerobic conditions, pH-controlled nutrient media; • Supplementation with prebiotic and probiotic formulation; • Sampling at 0, 5, 10, 24, 30 and 48 h.	• Cost-effectiveness of a reliable model to evaluate prebiotic and probiotic modulatory effects on elderly gut microbiota and immune responses.	• Potential therapeutic strategies for gut health in aging populations.	• Microbial shifts may not reflect in vivo dynamics due to model limitations.	[14]
	• Multiphase digestion simulation (oral, gastric, intestinal).	• Standardization for oral, gastric, and intestinal digestion conditions.	• Standardized protocol for simulating multicompartment food digestion.	• May not account for host–microbiome interactions.	[15]
	• Fecal inoculum from healthy and IBD-diagnosed donors; • The Simulator of the Human Intestinal Microbial Ecosystem (SHIME) with static configuration; • Supplementation with food additives; • Sampling at 72 h.	• Using a standardized protocol to assess the impact of food additives on microbial diversity and immune response to metabolites in bacterial communities obtained from healthy donors and IBD patients.	• Potential milestone in using a standardized protocol to compare healthy and pathological gut microbiota samples.	• Individual variability of microbiota	[16]
	• Inoculum: standard strains of *B. adolescentis, B. thetaiotaomicron, F. prausnitzii, R. inulinivorans*; • Anaerobiosis, physiological pH; • Sampling at 0–96 h.	• Novel in vitro combinations of microbial species based on co-occurrence network predictions.	• Understanding and design of synthetic community formulations.	• Reductionist approach; • Culture–dependent biases.	[17]
	• Fecal inoculum from healthy donors; • Anaerobic conditions, pH-controlled nutrient media; • Supplementation with *Chlorella pyrenoidosa* powder; • Sampling at 0, 6, 12, 24, and 48 h.	• Evaluation of polysaccharide fermentation, pH changes, and intestinal microbiota diversity modulation.	• Understanding the metabolism of polysaccharides from *Chlorella pyrenoidosa* powder during intestinal fermentation.	• Short-term analysis.	[18]
	• Fecal inoculum from adult pigs; • Anaerobic conditions; • Supplementation with *Fusarium* mycotoxins; • Sampling at 24 h.	• Evaluation of *Fusarium* mycotoxins biotransformation during intestinal fermentation. • Intestinal fermentation was simulated for different intestinal segments (duodenum, jejunum, caecum, colon, and rectum).	• An easier and less expensive method to obtain preliminary information on mycotoxin metabolism. • Could offer perspectives on the cytotoxic potential of the bacterial transformation products. • Could assist in designing further feeding trials.	• Short-term analysis.	[19]
	• Fecal inoculum from colonic active Crohn disease; • Continuous colonic model system (communicating vessels simulating intestinal segments); • Supplementation with rifaximin; • Sampling at 72 h.	• Evaluation of rifaximin-modulated changes in the bacterial diversity and metabolism; • Evaluation of genotoxicity, cytotoxicity, and chemopreventive potential of rifaximin.	• Studies on the effects of drugs (antibiotics) on gut microbiota.	• May not account for host–microbiome interactions.	[20]
	• Fecal inoculum from non-alcoholic steatohepatitis mice model; • Anaerobiosis; • Supplementation with omeprazole, phenacetin, midazolam, tolbutamide, metoprolol, and chlorzoxazone; • Sampling at 72 h.	• Evaluation of the biotransformation of different drugs by intestinal microbiota. • Evaluation of host–microbiota interactions.	• Studies on gut microbiota effects in drug metabolism.	• Host metabolism individual variability.	[21]
DFMs	• Fecal inoculum from healthy donors; • Anaerobic conditions, MiPro bacterial growth media (micro and macro quantitative); • Sampling at 0, 3, 6, 9, 12, 24, 36, and 48 h.	• Standardization of DFM culture media–MiPro. • Developing fecal-derived short-term microbial communities to evaluate bacterial growth and diversity. • Milestone in developing a culturing medium (MiPro) fit for both human and animal model-derived microbiota samples.	• Functional and compositional studies of intestinal microbiota.	• Limited host–microbiome interactions.	[22]
	• Fecal inoculum from suckling piglets (27 days old); • Dynamic anaerobic conditions based on peristaltic pumps (SHIME system); • Sampling at 4 weeks.	• Dynamic simulation of digestion using multichambered bioreactors. • Milestone in providing efficient simulation of mammalian gut structure and functions, including: peristalsis, culture media, pancreatic juice, bile, all the fermentation liquids, and constant pH.	• Complex studies involving the understanding of microbiota changes during digestion.	• Limited direct applicability to human microbiota.	[23]
	• Fecal inoculum from healthy donors; • Anaerobic conditions, CoMiniGut prototype; • Supplementation with inulin and lactulose; • Sampling at 24 h.	• Small–scale model for screening microbial fermentation processes.	• Rapid testing of gut microbiota activity.	• Static nature limits continuous flow simulation.	[24]
	• Fecal inoculum from healthy donors; • Dynamic anaerobic conditions based on peristaltic pumps (SHIME system); • Controlled oxygen gradients.	• Validated model for microbiota in the distal parts of the small intestine. • Milestone in standardizing the SHIME system for inoculation with human real intestinal content and feces.	• Enhanced microbiome research in small intestine models.	• Complex setup and maintenance.	[25]
CCMs	• Fecal inoculum from healthy donors and spondylitis patients; • Triple CCM: T84, LS-174 T, and THP-1 cell lines; • Anaerobiosis; • Sampling at 48 h.	• Milestone in establishing a triple CCM using 3 different human-originating cell lines and fecal inoculum. • Evaluation of inflammatory response to microbiota from healthy and spondylitis patients.	• Host–microbiota interactions	• Individual variability of microbiota and inflammatory response	[26]
	• Co-cultured intestinal and mast cell lines: Caco2, HT-29, and HMC-1.2 cell lines; • Supplemented with probiotic formulations: *L. rhamnosus LR 32, B. lactis BL 04, B. longum BB 536*; • Sampling at 24 h.	• Establishing a CCM to investigate host–microbiota interactions: inflammatory response of the intestinal barrier to probiotic formulation.	• Promising model to investigate therapeutic approaches for gut inflammatory diseases.	• Limited to specific cell types.	[27]
	• Fecal inoculum from mice; • Three-stage colon simulation (communicating vessels with mucosal beads); • Anaerobiosis; • Sampling at 48 h.	• Evaluating the digestion of xylitol using an in vitro colon simulation consisting of communicating vessels and mucosal beads.	• Promising model to investigate intestinal digestion of nutrients.	• Narrow scope on specific metabolites.	[28]
	• Fecal inoculum from mice; • Anaerobic fermentation of LPS or SGF−2; • Cell lines: human Caco-2 and murine macrophage RAW264.7; • Fermentation supernatant was added to co-cultured cell lines; • Sampling at 24 h.	• Examine modulatory effects of fucoidan and its fermentation products on bacterial diversity and inflammatory responses.	• Advances in dietary fiber and immune interactions research.	• Complex interplay between dietary components and immune responses.	[29]
	• Fecal inoculum from mice; • Cell lines: human embryonic kidney cells and human Caco-2 lines; • Fecal preps from tacrolimus-treated mice were added to co-cultured cell lines; • Sampling at 48 h.	• Evaluation of gut microbiota diversity changes following treatment with tacrolimus. • Evaluation of gut microbiota participation in tacrolimus metabolism. • Milestone in evaluating drug pharmacokinetics changes by gut microbiota.	• Studies of gut microbiota influence on drug metabolism. • Including gut microbiota pharmacokinetic potential in prediction algorithms for drug dosage.	• Individual variability of host and microbiota metabolism.	[30]
	• Mycotoxins hydrolysed by artificial digestive juices; • Fecal inoculum from healthy donors; • Cell lines: human Caco-2/TC7 cells; • Sampling at 48 h.	• Evaluation of mycotoxin hydrolysis by digestive juices, intestinal transportation through monolayers of cell cultures.	• Studies of mycotoxin metabolism during digestion. • Intestinal transportation of mycotoxins and their metabolites. • Microbiota metabolism of mycotoxins.	• Individual variability of host and microbiota metabolism.	[31]
GCMs	• Microdevices consisting of cell culture microchannels and vacuum chambers; • Microfluidic cultures of Caco-2 intestinal epithelial cells.	• Mechanical system mimicking intestinal peristalsis at physiological temperature. • Environmental conditions determine villi development in Caco-2 intestinal epithelial cells cultures in approx. 100 h.	• Studies bacterial overgrowth and inflammation mechanisms.	• Extensive resources needed.	[32]
	• Human colon monolayers obtained from human intestinal organoids; • Inoculation with *B. thetaiotaomicron* standard strain; • Both aerobiosis and anaerobiosis.	• Hos–microbiota interaction study: barrier integrity and adherence to mucus layer.	• Drug testing, microbiota-targeted therapies, and studying disease mechanisms.	• Maintaining stable bacterial cultures and compatibility with primary human cells.	[33]
OMs	• Mouse-originating small intestines; • Isolation of intestinal crypts; • Supplementation with bacterial products; • Inoculation with pathogenic bacterial species (*L. monocytogenes*).	• Allows efficient study of microbial colonization of intestinal epithelia and response to bacterial products. • Close-to-reality architecture. • Supports co-culture with immune cells to examine immune responses.	• Intestinal homeostasis and metabolic impairment during infection and inflammatory response. • Potential for developing personalized therapeutic approaches.	• Limited incorporation of other gut components (microbiota, vasculature).	[34]
	• Standardized human intestinal organoids; • Anaerobic conditions at apical surface; • Oxygen-controlled conditions.	• host–microbiome interactions; • Physiologically relevant oxygen environment; • Close-to-reality in vitro gut conditions.	• Host–microbiome interactions	• Difficult culture conditions could prevent reproducibility.	[35]
ISMs	• Computational simulation of cross-feeding between microbial species.	• Evaluation of bacterial community stability and metabolic interdependence.	• Understanding gut ecosystem resilience and response to microenvironmental changes.	• Translating in silico results to biological systems.	[36]
	• Bioinformatic analysis of metagenomic data.	• Identifies genes and enzymes responsible for flavonoid transformations.	• Dietary interventions and microbiome-targeted therapies	• Linking computational data to in vivo microbiota.	[37]
	• Computational docking and validation using antioxidant assays.	• Highlights bioavailability and efficacy of antioxidants.	• Nutraceutical development and therapeutic antioxidants research	• Translating findings into the physiological system	[38]
	• Simulation of metabolic impairments associated with pathological conditions.	• Describing metabolic pathways in dysbiosis	• Targeted microbiota modulation. strategies for therapeutic inventions.	• Validating computational predictions in clinical settings.	[39]

Abbreviations: SFM, static fermentation model; DFM, dynamic fermentation model; CCM, co-culture model; GCM, gut-on-a-chip model; OM, organoid model; ISM, in silico model; IBD, inflammatory bowel disease; TMA, trimethylamine; γ-BB, γ-butyrobetaine.

**Table 2:** Comparing the advantages and limitations of the main in vitro model types.

Model	General Design and Applications	Advantages	Limitations
SFMs [10,11,12,13,14,15,16,17,18,19,20,21]	• Short-term systems consisting of fecal or intestinal content samples cultured in controlled microenvironments (e.g., pH, temperature, aerobiosis). • Generally used to evaluate interactions between microbial communities and specific substrates (e.g., short-chain fatty acids).	• Easy setup, minimal equipment requirements. • Basic environmental conditions (e.g., pH, aerobiosis). • Suitable for routine, short-term studies, initial screening of microbiota diversity and responses.	• Peristalsis, mucus secretion, and dynamic nutrient flow could not be provided. • Short-term microbial communities.
DFMs [22,23,24,25]	• Extended period cultures of intestinal microbiota that benefit from replenishing resources. • Could be used to evaluate the microbiota responses to nutrient flow and microbial adaptation (e.g., dietary fibers, probiotic treatment, pH).	• Good simulations of intestinal transit and segmentation. • Suitable for long-term (up to several weeks) observation of microbial communities.	• Require multiple resources at high costs, specialized equipment, and advanced expertise. • Could not provide insights into the interactions between microbial communities and host tissues.
CCMs [26,27,28,29,30,31]	• More complex models that include microbiota and >2 cell types. • More accurate simulation of the intestinal environment. • Could be used to study the complex microbiota–intestine interactions (e.g., mucosal response, digestive functions, immunological response).	• Simulation of gut epithelial lining and inflammatory cells. • Allow the study of mucosal barrier integrity and the selection of specific microbial species for interaction studies.	• Complex microbial communities exhibit growth competition. • Dependent on anaerobiosis.
GCMs [32,33]	• Microdevices based on microfluid technology recreating intestinal physiological conditions. • Efficient in simulating peristaltic movements and nutrient flow. • Advanced models of simulated digestion allowing the study of complex physiological, metabolic, immunological, and pharmacological interactions with both intestinal epithelia and microbiota.	• Able to incorporate mechanical peristalsis and nutrient flow. • Enabling real-time analysis of complex microbiota–host interactions. • ∙	• Increased costs and significant resources to produce, operate, and read. • Small-scale devices are not suitable for high-throughput studies. • ∙
OMs [34,35]	• Self-organized stem cells-originating 3D structures resembling the organ architecture and functions. • Accurate replication of human intestine microstructures. • Efficient in providing insights into the microbiota modulation of development, function, and regeneration of the intestinal epithelium	• Complex architectures of gut lining. • Supporting advanced studies of host–microbiota interactions.	• The specific cell culture requirements prevent complex microbial community development.
ISMs [36,37,38,39]	• Computer-simulated models allowing the study of human microbiota behavior and interaction (e.g., predicting microbial interactions, microbial community diversity evolution). • Complex algorithms, databases, and mathematical models, with or without the involvement of artificial intelligence-based solutions.	• Fast simulations of microbial interactions and metabolic processes. • Not requiring physical resources.	• Predefined algorithms with limited degrees of complexity. • Need in vitro/in vivo validation.

SFMs—static fermentation models; DFMs—dynamic fermentation models; CCMs—co-culture models; OMs—organoid models; GCMs—gut-on-a-chip models; ISMs—in silico models.

## Data Availability

No new data were created.

## Supplementary Materials

The following supporting information can be downloaded at: https://www.mdpi.com/article/10.3390/medicina62030554/s1, File S1: PRISMA 2020 Checklist [92].

## Abbreviations

The following abbreviations are used in this manuscript:

MeSH	Medical Subject Headings
TMA	Trimethylamine
SFM	Static fermentation model
BFM	Dynamic fermentation model
SHIME	Simulator of the Human Intestinal Microbial Ecosystem
MiPro	croplate-based culturing model
CoMiniGut	Copenhagen MiniGut
CCM	Co-culture model
Caco2	Cancer coli 2
HMC	Human mast cell
LPS	Lipopolysaccharides
SGF-2	Silk Gland factor-2
GCM	Gut-on-a-chip model
OM	Organoid model
ISM	In silico model
IBD	Inflammatory bowel disease
CRC	Colorectal cancer
IBS	Irritable bowel syndrome
